# RETRACTED: Syah et al. A New Hybrid Algorithm for Multi-Objective Reactive Power Planning via FACTS Devices and Renewable Wind Resources. *Sensors* **2021**, *21*, 5246

**DOI:** 10.3390/s24144740

**Published:** 2024-07-22

**Authors:** Rahmad Syah, Peyman Khorshidian Mianaei, Marischa Elveny, Naeim Ahmadian, Dadan Ramdan, Reza Habibifar, Afshin Davarpanah

**Affiliations:** 1 Data Science & Computational Intelligence Research Group, Universitas Medan Area, Medan 20223, Indonesia; rahmadsyah@staff.uma.ac.id (R.S.); dadan@uma.ac.id (D.R.); 2 Department of Electrical and Computer Engineering, Babol Noshirvani University of Technology, Babol 47148-71167, Iran; peyman.khorshidianmianaei@gmail.com; 3 Data Science & Computational Intelligence Research Group, Universitas Sumatera Utara, Medan 20155, Indonesia; 4 W. P. Carey School of Business, Arizona State University, Tempe, AZ 85281, USA; naeim.dian@asu.edu; 5 Department of Electrical Engineering, Sharif University of Technology, Tehran 113651-1155, Iran; rhabibifar@alum.sharif.edu; 6 Department of Mathematics, Aberystwyth University, Aberystwyth SY23 3BZ, UK

The *Sensors* Editorial Office retracts the article, “A New Hybrid Algorithm for Multi-Objective Reactive Power Planning via FACTS Devices and Renewable Wind Resources” [1], cited above.

Following the publication, concerns were brought to the attention of the Editorial Office relating to the validity of the findings and the relevancy of a significant number of citations included within this article [1]. 

Adhering to our complaints procedure, an investigation was conducted by the Editorial Office and the Editorial Board which detected irregularities within the presented findings and confirmed the presence of a high number of citations that lacked sufficient relevance to this publication.

As the authors were unable to satisfactorily address these concerns, the Editorial Board have lost confidence in the integrity of the findings and have decided to retract this publication [1], as per MDPI’s retraction policy (https://www.mdpi.com/ethics#_bookmark30) (accessed on 15 July 2024).

This retraction was approved by the Editor-in-Chief of *Sensors*.

The authors did not agree with this retraction.
